# Correction: Stimulus Discrimination in Cerebellar Purkinje Neurons

**DOI:** 10.1371/journal.pone.0101376

**Published:** 2014-06-20

**Authors:** The PLOS ONE Staff

## Notice of Republication

This article was republished on April 21, 2014 to replace an incorrectly published version where an equation in the Materials and Methods section was omitted in the PDF and XML during the typesetting process. Please download this article again to view the correct version. The originally published, uncorrected article and the republished, corrected article are provided here for reference.

## Supporting Information

File S1 Originally published, uncorrected article.
(PDF)

File S2 Republished corrected article.
(PDF)
