# Supplementary material for: Correction: Stimulus Discrimination in Cerebellar Purkinje Neurons
Source: PLoS One. 2014 Jun 20;9(6):e101376. doi: 10.1371/journal.pone.0101376 (PMC4065103; doi:10.1371/journal.pone.0101376)
Supplement: File S2 — Republished corrected article. (PDF) [file pone.0101376.s002.pdf]

PLOS | ONE

Stimulus Discrimination in Cerebellar Purkinje Neurons

Katharina Reusch[1,2], Noah A. Russell[2], Tomas C. Bellamy[1]*

1 School of Life Sciences, University of Nottingham, Nottingham, United Kingdom, **2** School of Electrical and Electronic Engineering, University of Nottingham, Nottingham, United Kingdom

Abstract

Cerebellar Purkinje neurons fire spontaneously in the absence of synaptic input. Overlaid on this intrinsic activity, excitatory input from parallel fibres can add simple spikes to the output train, whereas inhibitory input from interneurons can introduce pauses. These and other influences lead to an irregular spike train output in Purkinje neurons *in vitro* and *in vivo*, supplying a variable inhibitory drive to deep cerebellar nuclear neurons. From a computational perspective, this variability raises some questions, as individual spikes induced by excitatory inputs are indistinguishable from intrinsic firing activity. Although bursts of high-frequency excitatory input could be discriminated unambiguously from background activity, granule neurons are known to fire *in vivo* over a wide range of frequencies. This would mean that much of the sensory information relayed through the cerebellar cortex would be lost within the random variation in background activity. We speculated that alternative mechanisms for signal discrimination may exist, and sought to identify characteristic motifs within the sequence of spikes that followed stimulation events. We found that under certain conditions, parallel fibre stimulation could reliably add a "couplet" of spikes with an unusually short interspike interval to the output train. Therefore, despite representing a small fraction of the total number of spikes, these signals can be reliably discriminated from background firing on a moment-to-moment basis, and could result in a differential downstream response.

Citation: Reusch K, Russell NA, Bellamy TC (2014) Stimulus Discrimination in Cerebellar Purkinje Neurons. PLoS ONE 9(2): e87828. doi:10.1371/journal.pone.0087828

Editor: Maurice J. Chacron, McGill University, Canada

Received October 29, 2013; **Accepted** December 30, 2013; **Published** February 5, 2014

Copyright: © 2014 Reusch et al. This is an open-access article distributed under the terms of the Creative Commons Attribution License, which permits unrestricted use, distribution, and reproduction in any medium, provided the original author and source are credited.

Funding: This work was funded by the UK Biotechnology and Biological Sciences Research Council (grant number: BB/J015660/1) and the Engineering and Physical Sciences Research Council (grant number: EP/H022112/1). The funders had no role in study design, data collection and analysis, decision to publish, or preparation of the manuscript.

Competing Interests: The authors have declared that no competing interests exist.

* E-mail: tomas.bellamy@nottingham.ac.uk

Introduction

Purkinje neurons are the principal output from the cerebellar cortex, forming a GABAergic connection to deep cerebellar nuclear (DCN) neurons. As with other classes of inhibitory neurons, Purkinje neurons exhibit spontaneous activity in the absence of synaptic input [1], which arises from the presence of resurgent Na^+ currents that sustain spiking under resting conditions [2]. Purkinje neurons can also undergo transitions to a "down" state where spontaneous spiking ceases [3–5]. Excursions between up and down states can be driven by climbing and parallel fibre inputs [6], but can also occur in the absence of input [3]. When inhibitory input to the Purkinje neurons from basket and stellate cells is blocked, spontaneous spiking becomes more regular [7], suggesting that inhibition serves to interrupt pacemaker activity, increasing the complexity of the spontaneous spike train structure.

This spontaneous activity raises questions about how sensory input into the cerebellar cortex affects the output from Purkinje neurons. At the simplest level of consideration, sensory input via granule cell parallel fibres will add spikes to the spontaneous activity in the output train, resulting in a transient increase in the rate of discharge to DCN neurons, and Khodakhah *et al.* have shown that Purkinje neurons linearly encode the strength of inputs into the frequency of spikes [8,9]. An alternative hypothesis advanced by De Schutter *et al.* is that afterhyperpolarisation following dendritic calcium influx introduces a pause that relieves the inhibitory output from the Purkinje neurons on downstream DCN cells [10]. All of these influences would depend to varying extents on the frequency, magnitude and pattern of parallel fibre input that a given sensory stimulus evokes. The frequency and complexity of parallel fibre firing rates *in vivo* also varies substantially [11–13], with some granule neurons firing in high-frequency bursts separated by periods of silence, and others firing more regularly at frequencies ranging from <0.4–10 Hz [12].

Viewed from the perspective of ongoing activity in an individual cell, this complexity imposes a problem of signal discrimination. Given variable background activity, can a transient change in spike frequency be definitely attributed to an input (and result in a different response in DCN neurons), rather than irregularity in spontaneous activity? This is particularly problematic with sparse or weak inputs, where stimulus-evoked events are rare and so greatly outnumbered by spontaneous events. If the Purkinje neuron acts as a filter in the microcircuit that passes high spike-frequency inputs [5] this is a moot question, but it would mean that any signals being conveyed by parallel fibres firing at rates less than or equal to background activity has no obvious consequence. Given the presence of such inputs [12] this seems inefficient, and so additional computational mechanisms for decoding low frequency connections may exist, which would allow them to be detected in the output train from Purkinje neurons as distinct from spontaneous spiking.

To address this uncertainty, we sought to identify which features of post-stimulus spike patterning best enabled discrimination between evoked responses and variable spontaneous spiking. We investigated the minimal requirements for reliable discrimination,

and the stimulation conditions necessary to meet these requirements. The results show that parallel fibre stimuli can be discriminated from spontaneous spikes when a characteristic "couplet" of spikes is evoked. In contrast, introduction of a post-stimulus pause into the spike train cannot allow discrimination of stimuli from spontaneous events, nor does it improve the accuracy of couplets in detecting stimulus events. We found no evidence for more complex spike motifs, and so argue that the short interval of post-stimulus couplets compared to background activity is sufficient to allow even low frequency inputs to be identified amongst the preponderance of spontaneous spiking events.

Materials and Methods

Ethics Statement

All experiments were performed in accordance with the guidelines set out in the code of practice for humane killing under Schedule 1 of the UK Home Office Animals (Scientific Procedures) Act 1986, and were approved by the University of Nottingham Animal Welfare and Ethical Review Body.

Slice Preparation

16–25 days old Wister rats (male and female) were killed by decapitation after cervical dislocation. The vermis of the cerebellum was removed quickly and placed into ice cold buffer containing (mM): NaCl (126), KCl (3), NaH_2PO_4 (1.2), $NaHCO_3$ (25), glucose (15), $MgSO_4$ (3), gassed with carbogen (95% O_2/5% CO_2). Sagittal and transverse 300 μm slices were prepared using a Leica VT 1000S vibratome containing ice cold buffer as above. Slices were transferred into a recovery chamber containing buffer as above but with 2 mM $MgSO_4$ and 2 mM $CaCl_2$ at 32°C for 1 h. Thereafter slices were kept at room temperature (22°C–24°C) for >30 min before experiments were carried out.

For each experiment one slice was transferred into an immersion chamber of a upright microscope (Nikon E600FN, 40× phase objective) under continuous perfusion with buffer as above (containing 2 mM $MgSO_4$ and 2 mM $CaCl_2$). NBQX (2,3-dihydroxy-6-nitro-7-sulfamoyl-benzo[f]quinoxaline-2,3-dione di-sodium salt) and bicuculline methiodide were from Tocris (Bristol, UK), and added to the bath solution at the concentrations indicated in the text.

Electrophysiological Recordings

Patch electrodes for loose cell attached recordings were filled with bath solution, and had a pipette resistance of 1–3 MΩ. Recordings were carried out with an Axopatch 200B amplifier (Axon Instruments, Sunnyvale, CA, USA) and with a seal resistance of 20–40 MΩ. For whole cell current clamp recordings the internal solution contained (mM): KCl (150), HEPES (10), $MgCl_2$ (4.6), ATP (4), GTP (0.4), EGTA (0.05), pH 7.3, 300 mOsm. Recordings were low pass filtered at 5 kHz, and sampled at 25 kHz with a micro1401 A/D convertor (CED; Cambridge, UK).

Parallel fibres were stimulated in sagittal slices, and granule cell somata were stimulated in transverse slices ≥100 μm distant from the recorded cell. Stimulation electrodes were filled with bath solution and had pipette resistances between 1–3 MΩ. Stimulation was carried out using a constant current isolated stimulator (DS3, Digitimer; Welwyn Garden City, UK) at stimulation intensities ranging from 5–100 μA. Where random stimulation patterns were applied, the protocol was created in Matlab (Mathworks, USA, Version 8.1), with intervals sampled from a Poisson distribution with a mean of 0.2 or 1 Hz.

Analysis and Simulations

All experimental data were analysed offline using Spike2 (CED, Cambridge, UK). Statistical tests were carried out in Prism (Graphpad, La Jolla USA, Version 6.0), using paired *t* test, or multiple comparison ANOVA test (with Tukey correction), as indicated in figure legends.

Simulated data were created using Matlab by sampling from a Gaussian distribution with mean 50 ms interval and standard deviation of 12.5 ms. Spikes were added to these simulated trains with regular 1 Hz frequency, or with random intervals sampled from Poisson distribution with a mean of 1 Hz.

To quantify the difference (D) between two ISI distributions a distribution-difference method was used to determine the Euclidean distance between the populations (see [14]). Histograms were normalised and the difference between n bins calculated, resulting in values of D ranging between 0 (=identical distributions) and 1 (=maximal difference, no overlap):

$$D = \frac{1}{2}\sum_{i=1}^{n} |A_i - B_i|$$

Couplet motif analysis was performed in Matlab, by varying the interval length between the two spikes used to define a couplet, over the range 0–50 ms. Once the couplet interval length was defined, the record of all interspike intervals was scanned to find motif matches. Motifs were then categorised as true positives if the first spike of the detected couplet occurred <50 ms after a stimulus event. False positives were any couplets that were detected outside of this post-stimulus window.

Results

Addition of Single Spikes to Spontaneous Activity

Many previous reports have shown that stimulation of parallel fibre inputs to Purkinje neurons results in an increase in the maximum firing rate of the cells. In theory, this result is self-evident, as the addition of extra spikes to an existing spike train will inevitably decrease the interval between preceding and following spikes, resulting in a transient increase in maximum rate. Under conditions where feedforward inhibition is blocked, the number of spikes and the maximum frequency both increase linearly with stimulus strength [5], but when feedforward inhibition is present, the number of spikes added can be reduced to a single spike [8,15]. In principle, this would still increase maximum frequency, but when imposed on a variable background spike train, the increase may be hard to identify within the existing variance.

To investigate the impact of adding a single spike on the distribution of spike intervals, we simulated data based on published values for spontaneous activity in Purkinje neurons. With a mean spontaneous firing rate of 20 Hz, the addition of single spikes at 1 Hz (in red, Fig. 1A) introduced a leftwards skew to the distribution of interspike intervals (ISI; Fig. 1B). A closely similar result was obtained if the simulated input arrived randomly, rather than regularly (Fig. 1B). This result confirms that simply through addition of a spike that bisects an existing interval in the spontaneous spike train, a new population of shorter-than-average intervals results (N.B. no refractory period was incorporated into the simulation, so this is the best case scenario for introducing short intervals). This population includes both pre- and post-stimulus intervals, as the cell would not have an independent measure of stimulus time, so an additional spike arriving soon after an existing spike, or arriving immediately

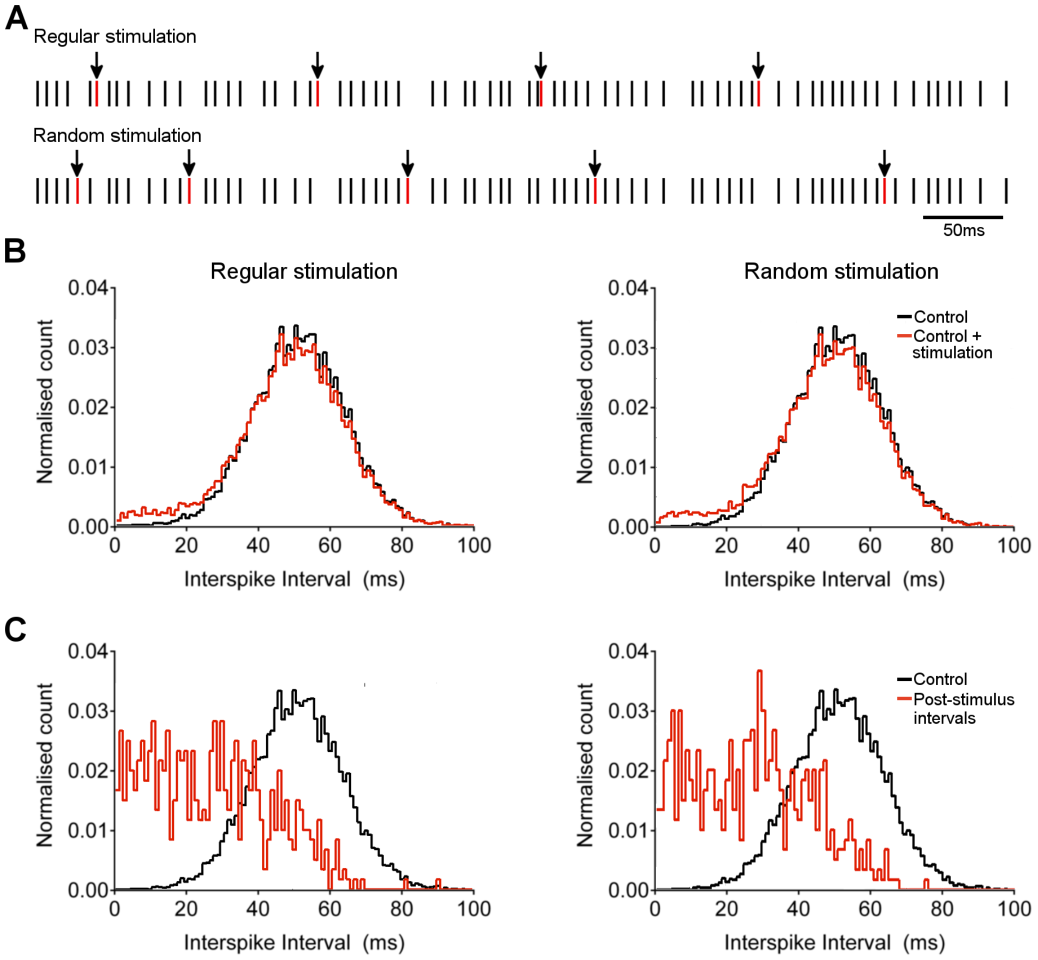

Figure 1. Addition of single spikes to a spike train. A) A spike train (black lines) was generated by sampling from a Gaussian distribution of interspike intervals (mean = 50 ms, SD = 12.5 ms), to which additional single spikes were added to simulate a stimulus event (red lines, arrows) at a regular frequency of 1 Hz, or randomly with a mean of 1 Hz (see Methods for more details). No refractory period was incorporated. B) Normalised distributions of interspike intervals before (black line) and after (red line) addition of single spikes to the train, with regular (first panel) and random (second panel) stimulus times. C) Distribution of post-stimulus intervals (red line) overlaid on distribution in the absence of stimulation (black line). Note appearance of a population of shorter than average intervals due to bisection of existing intervals.
doi:10.1371/journal.pone.0087828.g001

before an existing spike could equally result in a shorter that average interval.

Segregating the population into spontaneous and evoked spikes (Fig. 1C), makes it clear that while a short ISI is predictive of a stimulus event, many false negatives would occur – that is, many stimulus-evoked events will be indistinguishable from background activity. So, while the influence of stimuli can be seen at the population level, for any given spike it is not possible to determine whether it occurred in response to a stimulus, or spontaneously.

Excitatory and Inhibitory Input to Purkinje Neurons

Loose cell-attached recordings were used to investigate the pattern of spontaneous and evoked spiking in Purkinje neurons in *in vitro* slices of rat cerebellum. Under control conditions, spontaneous spiking was observed with a pattern similar to previous reports [1]: ISIs showed an approximately Gaussian distribution with a rightwards skew, with a tail of longer intervals that correspond to "down" states where spiking ceased (Fig. 2A). Addition of the $GABA_AR$ antagonist bicuculline (20 μM) to the bath solution resulted in an increase in the total number of spikes, a leftwards shift in mean ISI, and a reduction in the length and number of down periods (Fig. 2A), indicating that tonic inhibitory transmission acts to suppress background activity. Addition of the AMPA/kainate receptor antagonist NBQX (10 μM) to the bath had little impact on the distribution of observed ISIs (Fig. 2A), suggesting that spontaneous release from excitatory terminals does not significantly influence background spiking in Purkinje neurons.

Stimulation of parallel fibres in the molecular layer at an intensity of 25 μA resulted in the generation of one or two spikes in the Purkinje neuron, typically within 20 ms of the stimulus (Fig. 2B,C). Blockade of inhibitory transmission with bicuculline increased the number of post-stimulus spikes (Fig. 2B,C,D), and blockade of excitatory transmission with NBQX eliminated stimulus-evoked spikes (Fig. 2B,C,D). Again, this is consistent with earlier reports [8,15] and confirms that under control conditions, where feedforward inhibition is intact, parallel fibre inputs add only a single or couple of spikes to the train of background activity.

Probability of Evoking More than One Spike

As shown in figure 1, the addition of a single evoked spike to spontaneous activity will not reliably allow stimuli to be

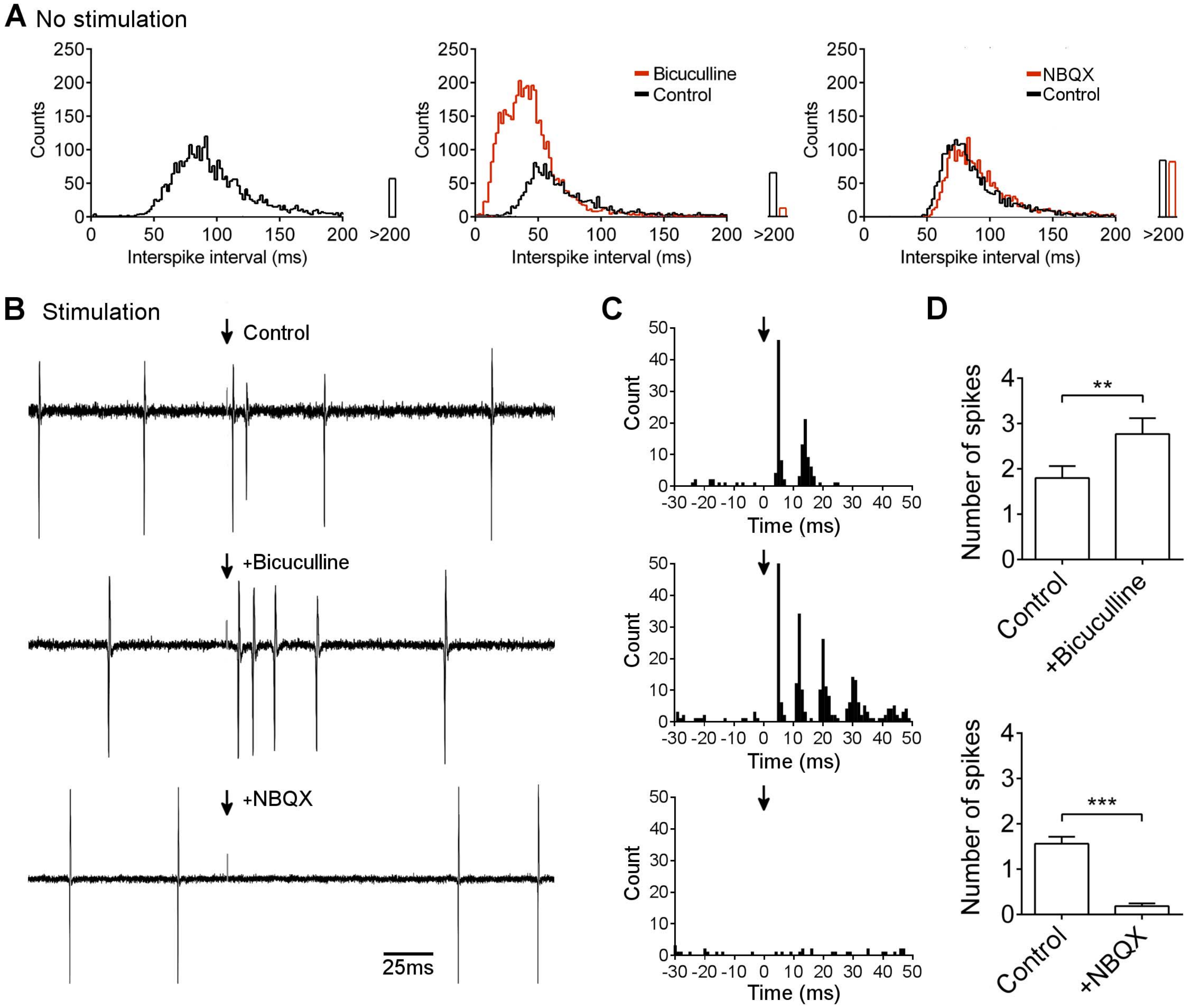

Figure 2. Effect of excitatory and inhibitory inputs on interspike interval. A) Distribution of spontaneous interspike intervals recorded over 10 min from a representative Purkinje neuron. Intervals greater than 200 ms in duration are pooled as a single population (bar graph to right of panel). First panel shows untreated cell, second panel shows a cell before (black line) and after (red line) 10 min incubation with 20 μM bicuculline, third panel shows a cell before (black line) and after (red line) 10 min incubation with 10 μM NBQX. B) Representative loose cell attached recordings from Purkinje neurons during stimulation of parallel fibre input in the molecular layer at 25 μA (arrow). Top trace shows untreated cell, middle trace shows cell incubated with 20 μM bicuculline for 10 min, lower trace shows cell incubated with 10 μM NBQX for 10 min. C) Post-stimulus histograms showing cumulative number of spikes from cells in panel B over 10 min of stimulation at 0.2 Hz. D) Aggregate data for mean ± s.e.m. number of post-stimulus spikes within 50 ms of stimulation before and after addition of bicuculline (top panel) or NBQX (bottom panel). n = 8 cells. **p = 0.0061 and ***p = 0.0003 (paired *t* test).
doi:10.1371/journal.pone.0087828.g002

discriminated from background activity. In contrast, the addition of two spikes to the train may be sufficient to allow discrimination, if the pattern of such a pair was characteristic for stimulus evoked events and rarely observed spontaneously. To investigate the conditions under which more than one post-stimulus spike was added, we varied the strength, position and number of stimuli imposed on the granule neuron input.

Increasing stimulation intensity in the molecular layer increased the probability of evoking two spikes (Fig. 3A,C,D). All stimulus strengths were significantly different from control (0 μA – where the record is marked but no stimulus applied), and increasing stimulus intensities between 5 and 50 μA significantly increased the mean number of spikes recorded (Fig. 3D). This result was observed regardless of whether cells were recorded in loose cell-attached or whole cell mode (Fig. 3A), indicating that recording configuration does not significantly affect excitability. The immediate interval between spikes has previously been reported to be linearly related to input strength [8]. We therefore examined the effect of stimulus intensity on immediate interval in those cases where two spikes were generated. Although there was a downward trend in mean interval, the dynamic range was small compared to variation from stimulus to stimulus, and no statistically significant differences were found except between the lowest (5 μA) and highest (75 and 100 μA) stimulus intensities (Fig. 3E,F).

In recent years, the physiological relevance of stimulating bundles of parallel fibres in the molecular layer has been

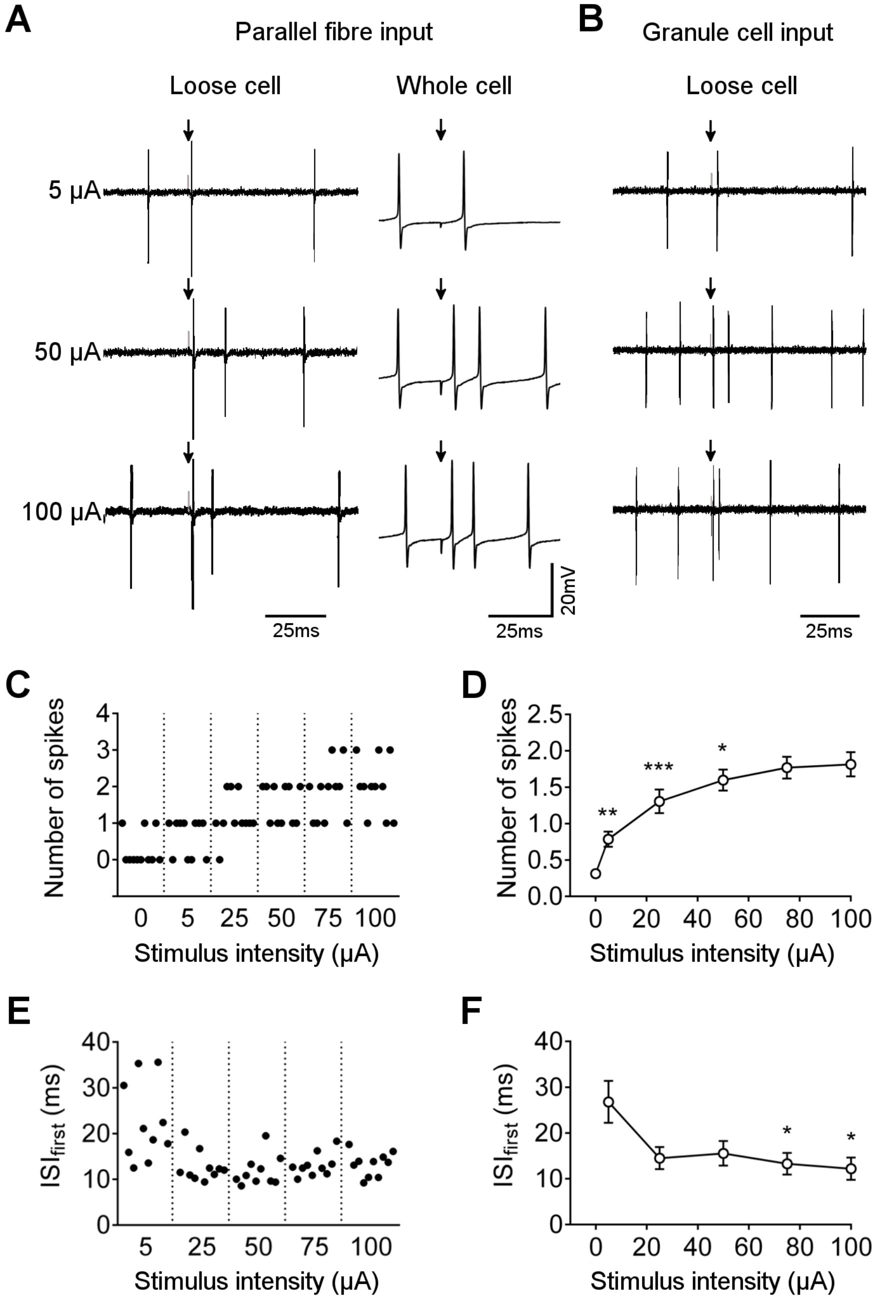

Figure 3. Effect of stimulus intensity on number of post-stimulus spikes. A) Loose cell (left panels) and whole cell (right panels) recordings from representative Purkinje neurons during stimulation in the molecular layer (arrow) at increasing intensities (as indicated). B) Loose cell recordings from representative cells during stimulation in the granular layer, at the same intensities as shown for molecular layer. C) Raster plot of number of post-stimulus spikes for 10 representative responses at each stimulus intensity. Note that 0 μA corresponds to sampling from the record in the absence of stimulus but with the same frequency as during stimulation, to give a measure of the probability of one or more spikes occurring within the sampling window by chance. D) Mean ± s.e.m. number of post-stimulus spikes against stimulus intensity (n = 15 cells). **p = 0.0053, ***p = 0.0009, *p = 0.0383, for comparison of mean to preceding (lower) stimulus intensity. Multiple comparisons ANOVA with Tukey's test for pairs (adjusted p values). E) Raster plot of 10 representative interspike intervals, for first pair of spikes, at each stimulus intensity. F) Mean ± s.e.m. of first interspike interval against stimulus intensity (n = 15 cells). *p<0.02 compared to 5 μA. Multiple comparisons ANOVA with Tukey's test for pairs.
doi:10.1371/journal.pone.0087828.g003

questioned [16], with stimulation in the granular layer to give a distributed parallel fibre input being considered more comparable to *in vivo* patterns of activity. When stimulating the granular layer in this way, the same result was observed: increasing stimulation strength increased the probability of evoking two spikes (Fig. 3B).

In addition to this spatial summation through increased recruitment of parallel fibres, we tested for temporal summation. Applying a single stimulus at 25 μA to either molecular or granular layer did not reliably evoke two spikes, but applying a short sequence of three stimuli at 100 Hz significantly increased the probability of evoking two (and occasionally more) spikes

(Fig. 4A–D). For some cells, the stimulus train evoked a pair of spikes only after the last stimulus (Fig. 4A), suggesting that temporal summation was needed to reach the firing threshold, but once reached, multiple spikes were evoked. This result was reliably observed in loose cell and whole cell configurations, and with stimulation in the granule cell layer (Fig. 4A,B). In other cases, spikes were generated during the stimulus train and the final stimulus resulted in two or more additional spikes (Fig. 4A,B). This result shows that some connections are able to relay input stimuli with high probability, and so the spike output pattern will follow the input pattern, but other connections do not directly relay input signals. Instead, the final output is a pair of spikes similar to that generated by a single stimulus of higher intensity.

Finally, we tested whether the resting state of the Purkinje neuron affected the probability of evoking more than one spike. Purkinje cells exhibit bistability, and spontaneously switch between "up" (repetitive firing) and "down" (silent) states, correlating with shifts in the resting membrane potential near the action potential threshold [17]. We investigated how resting state would influence the number of evoked spikes in cells that were stimulated to yield single and double spikes with approximately equal probability. Down states were defined as periods of >1 s without observed spikes preceding the stimulus, and up states as periods of frequent firing with a spike occurring <100 ms before the stimulus. There was no apparent effect of resting state on probability of evoking two spikes (Fig. 5A,B). In either up or down states, single spikes and double spikes were equally likely to occur, and there was no significant difference in mean number of spikes between the two conditions ($p = 0.147$, paired t test; $n = 6$ cells).

Collectively, these results indicate that either strong or repetitive stimulation of parallel fibre inputs, regardless of the spatial distribution of inputs or the resting state of the Purkinje neuron, results in an increased probability that two spikes will be added to the output train.

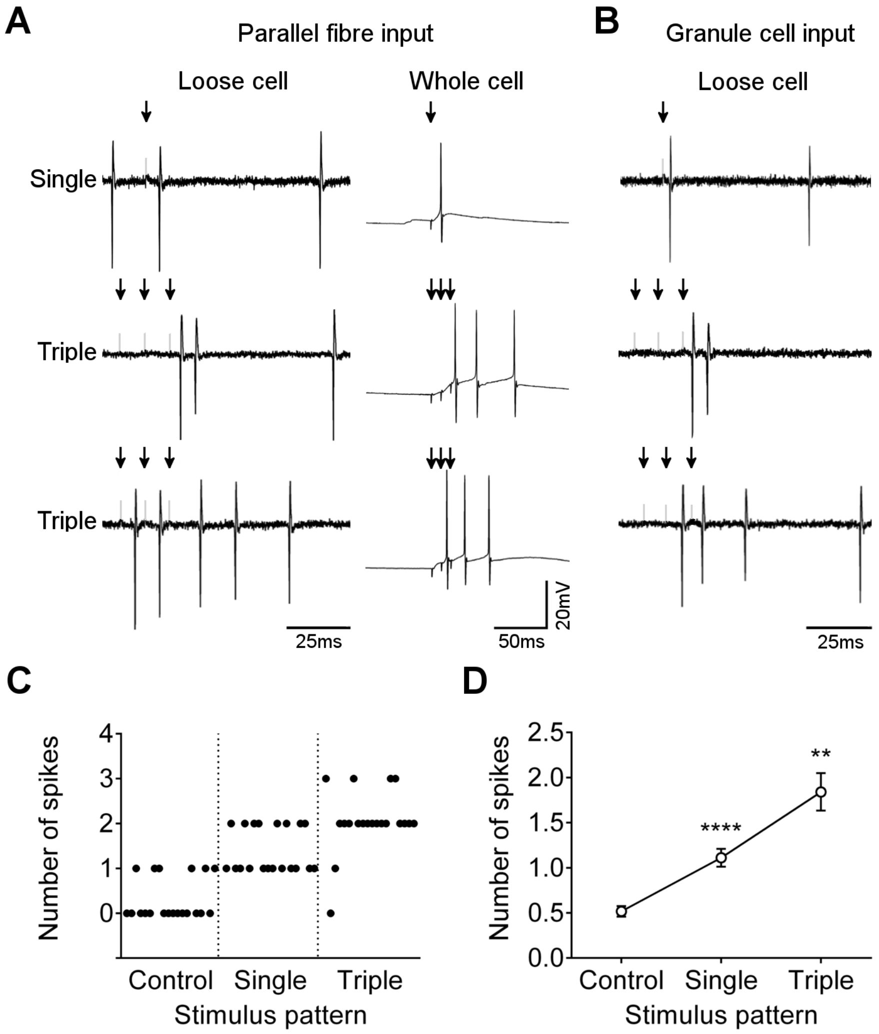

Figure 4. Effect of stimulus number on number of post-stimulus spikes. A) Loose cell (left panels) and whole cell (right panels) recordings from representative Purkinje neurons during stimulation at 25 µA in the molecular layer (arrow) with a single pulse (upper trace), or three pulses with 10 ms interval (lower traces). Lower two traces show different outcomes observed when spikes follow the final stimulus (middle trace) or when spikes are added during and after the stimulus train (lower trace). B) Representative loose cell attached recordings under the same conditions as panel A, but with stimulation in the granular layer. C) Raster plot of number of post-stimulus spikes for 20 representative responses during no stimulation, stimulation with a single pulse, or stimulation with 3 pulses. D) Mean ± s.e.m. number of post-stimulus spikes with single or triple pulses (note that only spikes that follow the end of the stimulus train are counted). ****$p < 0.0001$ compared to control, **$p = 0.002$ compared to single pulse. Multiple comparisons ANOVA with Tukey's test for pairs (adjusted p values).
doi:10.1371/journal.pone.0087828.g004

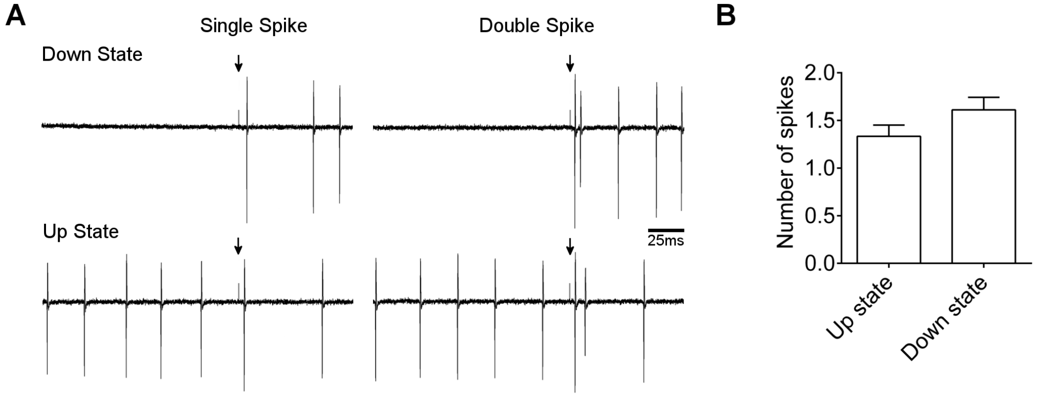

Figure 5. Effect of resting membrane state on number of post-stimulus spikes. A) Loose cell recordings from cells showing single post-stimulus spikes (left traces) or double post-stimulus spikes (right traces), evoked from down state (upper traces) or up state (lower traces). B) Mean ± s.e.m. number of post-stimulus spikes from cells in up or down states (n = 6). No significant difference between up and down state was detected: p = 0.084, paired *t* test.
doi:10.1371/journal.pone.0087828.g005

Post-stimulus Pause

We next examined the effect of these different stimulation parameters on the post-stimulus pause. Increasing stimulation intensity resulted in the appearance of a gap in raster plots (Fig. 6A), which followed the generation of one or two evoked spikes. This result is similar to previous reports [10,18], and corresponds to a pause in Purkinje neuron discharge, attributable to afterhyperpolarisation.

The mean length of the pause (where pause length is defined as the longest interval – ISI_{max} – beginning within 50 ms of the stimulus event) did not vary significantly with stimulation intensity (Fig. 6A,C,D), or with the distribution of inputs (Fig. 6A). Stimulation with a train of three stimuli at 100 Hz decreased the mean pause length compared to a single stimulus (Fig. 6B,E). Under all stimulation conditions, the post-stimulus pause (ISI_{max}) was not statistically significantly different from mean ISI_{max} in

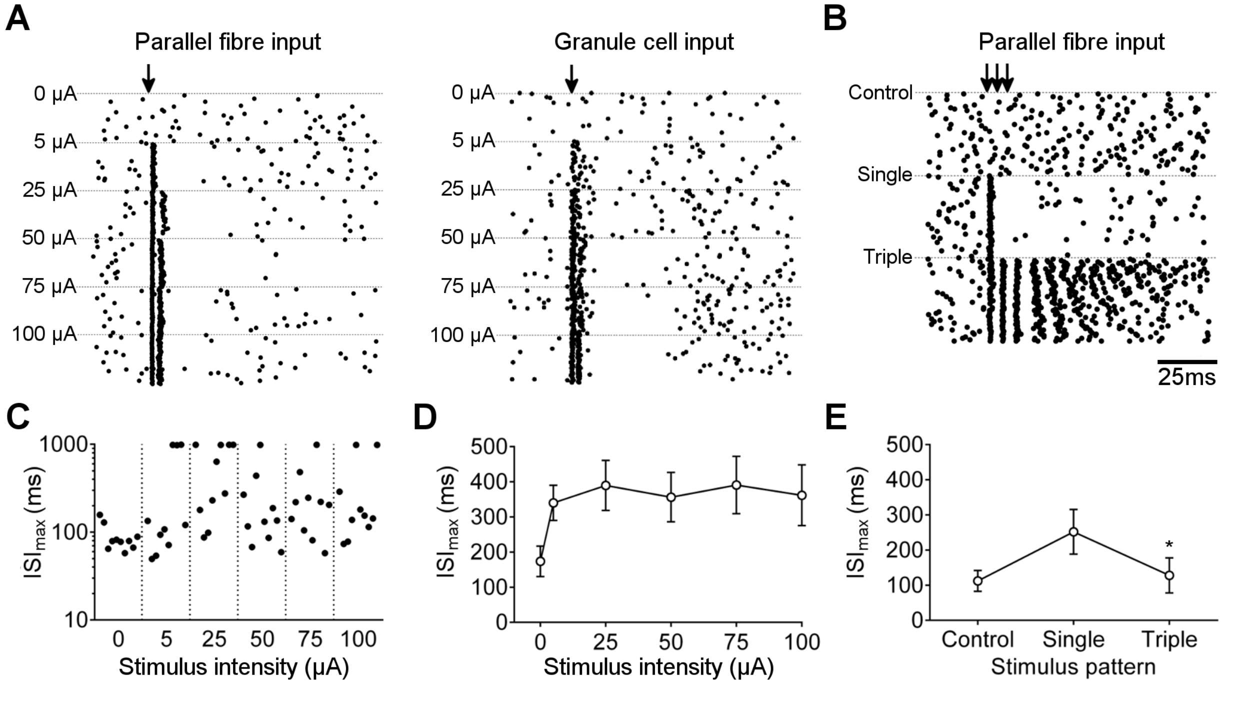

Figure 6. Effect of stimulus intensity on post-stimulus pause. A) Raster plot of 60 stimuli (arrow) at 0.2 Hz, with increasing stimulus intensity (0–100 μA as indicated), in the molecular layer (left panel) and granular layer (right panel). Note apparent gap in record post-stimulation. B) Raster plot as for panel A, with stimulation at 25 μA with single or triple pulse with 10 ms interval (as indicated). C) Raster plot of maximum post-stimulus interval (initiated within 50 ms of stimulus) for 10 representative responses at each stimulus intensity. Intervals of 1 s correspond to sweeps where no spikes were detected before the next stimulus. D) Mean ± s.e.m. maximum post-stimulus interval against stimulus intensity. No significant differences (all p values >0.05) were found between stimulus intensities or unstimulated cells (n = 16; Multiple comparisons ANOVA with Tukey's test for pairs). E) Mean ± s.e.m. maximum post-stimulus interval for unstimulated cells, and single and triple pulse stimuli (n = 17). *p = 0.042, multiple comparisons ANOVA with Tukey's test for pairs (adjusted p value).
doi:10.1371/journal.pone.0087828.g006

unstimulated cells (i.e. where the record was marked and subsequent spikes analysed, but no stimulus applied, to measure the occurrence of pauses during chance sampling from a spontaneous spike train).

These results suggest that despite a post-stimulus pause being noticeable in raster plots, it does not differ significantly in length from spontaneous pauses observed in unstimulated cells.

Distribution of Interspike Intervals for Spontaneous and Evoked Events

The pattern of two spikes, with or without a pause, evoked by stimulation may be sufficient to allow discrimination from background spiking, if the pattern does not occur commonly during spontaneous firing in unstimulated cells. To test this hypothesis, we compared the distributions of the first and the longest ISIs after stimulation, with the distribution of ISIs in the absence of stimulation.

Parallel fibres were stimulated at either 0.2 Hz or 1 Hz (Fig. 7A). Interspike intervals were then sorted into three populations: spontaneous events uncorrelated with stimuli, the first interval post-stimulation, and the longest interval (within 50 ms) post-stimulation; that is, spontaneous spikes, evoked spikes and pauses.

Under these conditions, post-stimulus spikes were a very small fraction of the total population (Fig. 7B), which illustrates the challenge of distinguishing this signal from background activity. For the first ISI, the events were strikingly shorter than for background activity, with little overlap between the populations (Fig. 7B). In contrast, the population of maximum ISIs (pauses) could not be clearly distinguished from background activity; the distribution of ISI_{max} appeared to be a subpopulation of the larger set (Fig. 7B).

To quantify the difference between these populations, we normalised the distributions and used a subtraction metric (see Methods, and [14]) as a measure of the Euclidean distance between the populations (D). With this measure, 0 corresponds to perfect overlap, and 1 corresponds to no overlap. For comparison of first ISI to spontaneous spikes, mean $D = 0.93$ at 0.2 Hz and 0.97 at 1 Hz (Fig. 7C). For comparison of post-stimulus pauses to spontaneous intervals, mean $D = 0.63$ at 0.2 Hz and 0.39 at 1 Hz (Fig. 7C). This analysis confirms that the immediate interval of

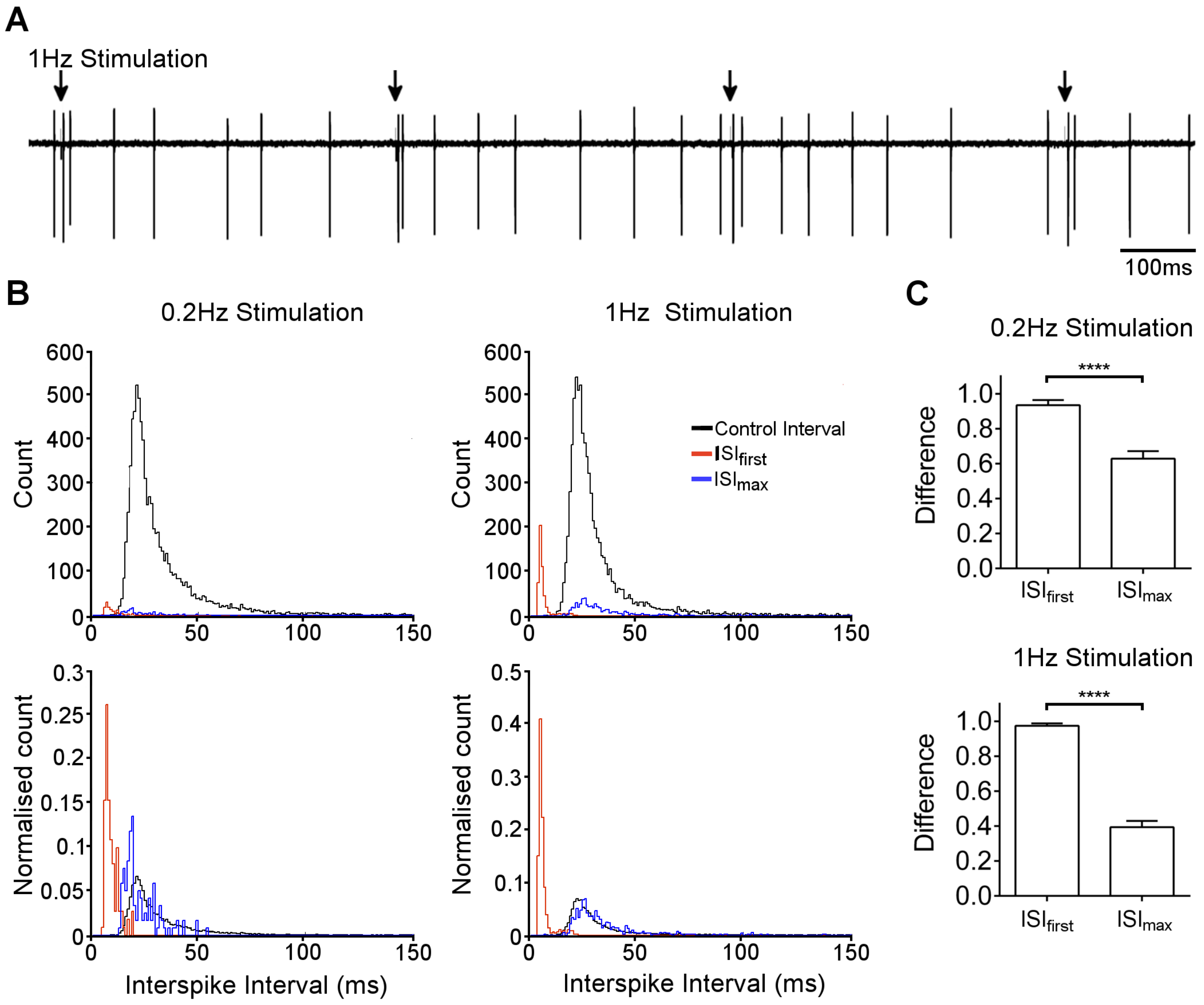

Figure 7. Distribution of post-stimulus intervals. A) Loose cell attached recording during regular stimulation at 1 Hz (arrow). B) Distribution of first post-stimulus interval (ISI_{first}, red line), maximum post-stimulus interval (ISI_{max}, blue line) and intervals uncorrelated with stimulus (black line). Left panels show representative cell stimulated at 0.2 Hz, right panels show representative cell stimulated at 1 Hz. Upper panels show total counts over 10 min, lower panels show populations normalised to AUC = 1. C) Mean ± s.e.m. difference between first interval (ISI_{first}) and maximum interval (ISI_{max}) using a distribution-difference metric (see Methods for details). ****$p<0.0001$, unpaired t test.
doi:10.1371/journal.pone.0087828.g007

post-stimulus pairs is clearly distinguishable from the range of intervals observed during spontaneous activity, but the post-stimulus pause is not.

Post-stimulus Spike Train Structure

Comparison of the overlap of spontaneous spike ISI histograms with the first and longest intervals post-stimulus may overlook detail in the post-stimulus spike train that is not obvious from simple visual inspection. It also only applies for stimuli that reliably evoked pairs of spikes and/or pauses. We next adopted a different approach to analysis, by plotting the post-stimulus intervals against one another at increasing numbers of dimensions, to investigate whether there was additional correlational structure within the pattern of post-stimulus spikes. This approach should reveal whether distinctive patterns emerge as multispike "motifs" that are not evident from investigating single intervals; a characteristic sequence of intervals may allow discrimination even if any one of the intervals could be observed spontaneously.

For cells routinely exhibiting two or more post-stimulus spikes, the first ISI gave clear discrimination from spontaneous activity, as previous described (Fig. 8A). Plotting the first ISI against the second ISI yields a two dimensional plot (Fig. 8A), and shows that the second ISI can adopt a wide range of values, as post-stimulus events are spread along the y axis. A cluster of events with short first and second intervals corresponds to triplets of post-stimulus spikes. Similarly, plotting the first three ISI values on a 3D plot (Fig. 8A), reveals a range of values that the third ISI can adopt, and again, the post-stimulus data is segregated from the spontaneous data only along the first ISI axis. This shows that first ISI alone is sufficient for signal discrimination, and that subsequent ISIs are independent of the presence of a stimulus (except for rare cases in which triplets or quadruplets result in a sequence of short ISIs).

For cells that exhibited either a single stimulus-evoked spike or variable numbers of spikes, a subpopulation of ISIs showed a similar peak at intervals less than spontaneously occurring ISIs, but the larger proportion of post-stimulus ISIs overlapped the spontaneous events (Fig. 8B,C). Furthermore, plotting the first ISI against the second and third ISIs did not reveal any additional structure to the pattern of spikes: the post-stimulus spikes do not differ from the spontaneous events on any axes, except for the subpopulation of short first intervals.

Collectively, these results show that the only element of the post-stimulus spike structure that allows discrimination from background activity is the first post-stimulus interval, when a pair of spikes is evoked with a strikingly short ISI. This pair of spikes can be considered as a "couplet" motif, characteristic of a stimulus event, and rarely observed spontaneously.

Predictive Power of Post-stimulus Couplets

To test the efficiency of the couplet motif for recognising stimulus-evoked signals within an overabundance of background activity, we recorded from Purkinje neurons stimulated at a frequency of 1 Hz. We tested the efficiency of the motif in three representative cells exhibiting mean post-stimulus spikes ranging from 1.04 to 2.85 (Fig. 9A).

To determine the optimal conditions for stimulus matching, we varied the interval length used to define a couplet from 0 to 50 ms. For each cell, we measured the number of stimulus events that were correctly detected (true positives) and the number of couplet motifs that were identified when a stimulus had not occurred (false positives). In all cells, true positives began to be detected when couplets had short intervals (5–10 ms), and the number of such "hits" rose to a plateau, indicating that the success in detecting stimuli did not improve substantially as the interval between the spikes in the couplet increased (Fig. 9B). In contrast, false positives began to appear when the interval length used to define the couplet motif increased beyond approximately 20 ms. The number of false positives increased dramatically as interval increased further, as once the couplet interval length increases to values commonly observed in spontaneous activity, the ability to discriminate is lost.

As a final note, in the cell with mean post-stimulus spike number of 2.85, the number of true positives actually exceeds the total number of stimuli (dashed line in Fig. 9B). This is because the algorithm recognises a triplet of spikes as two couplets, and so this would be assigned as two stimuli. Whether such triplets correspond to a separate motif, or whether any number of short intervals is sufficient for stimulus recognition is unclear.

Collectively, these results show that there is an interval "window" ranging from around 5–20 ms within which a couplet motif can be used to successfully detect stimulus events without appreciable error. When cells consistently exhibit post-stimulus couplets, stimuli are readily detected with high accuracy. In cells exhibiting post-stimulus means closer to 1 spike, the motif still allows the accurate detection of the minority of stimuli that result in short intervals. As the probability of evoking a couplet increases – with increased stimulus intensity or temporal summation (Figs. 3 and 4) – the accuracy of stimulus detection will therefore increase.

Discussion

The pattern of firing in cerebellar Purkinje neurons is subject to many influences. Intrinsic firing is overlaid with excitatory inputs that vary in firing frequency from <0.4 to 1000 Hz [12], and inhibitory inputs that form feedforward and feedback loops [19]. How the output from the Purkinje neuron encodes the strength and pattern of inputs has been much debated, with leading hypotheses being linked to both bursts and pauses in output [9,10]. We have investigated this microcircuit from a tangential perspective: can sparse, phasic input signals be discriminated from a large degree of tonic background firing? To address this question, we sought to identify structure in the pattern of the post-stimulus spike train that could act as a motif that would be sufficient to allow signal detection.

The main conclusion from this analysis is that a pair of spikes with an interspike interval that is substantially shorter than spontaneous events gives a high degree of success in identifying stimulus events. This "couplet" motif represents an effective mechanism for signal discrimination from background activity. Analysis of the interrelation of post-stimulus spike intervals indicated that no additional structure was evident in the train – for example, the sequential pattern of a single spike with shorter than average ISI followed by a pause of longer than average ISI may have been predicted from earlier results, but this would have given rise to a distinct cluster in 2D and 3D plots of intervals (Fig. 8). No such clustering was evident, and only the first interval offered clear separation from the intervals of spontaneous spikes.

A virtue of this couplet mechanism for "marking" the spike train with a stimulus-selective pattern is the economy of the motif, both from the perspective of cellular energetics, and for the number of motifs that can be incorporated in a given period of time (in contrast to longer pauses). It also benefits from a high timing precision, where the motif is correlated closely in time with the stimulus event. Furthermore, if, as hypothesised, the maximum rate of firing encodes the strength of input [9], two spikes are sufficient in principle to encode both the occurrence of a stimulus and its strength. In our data, this relationship did not appear

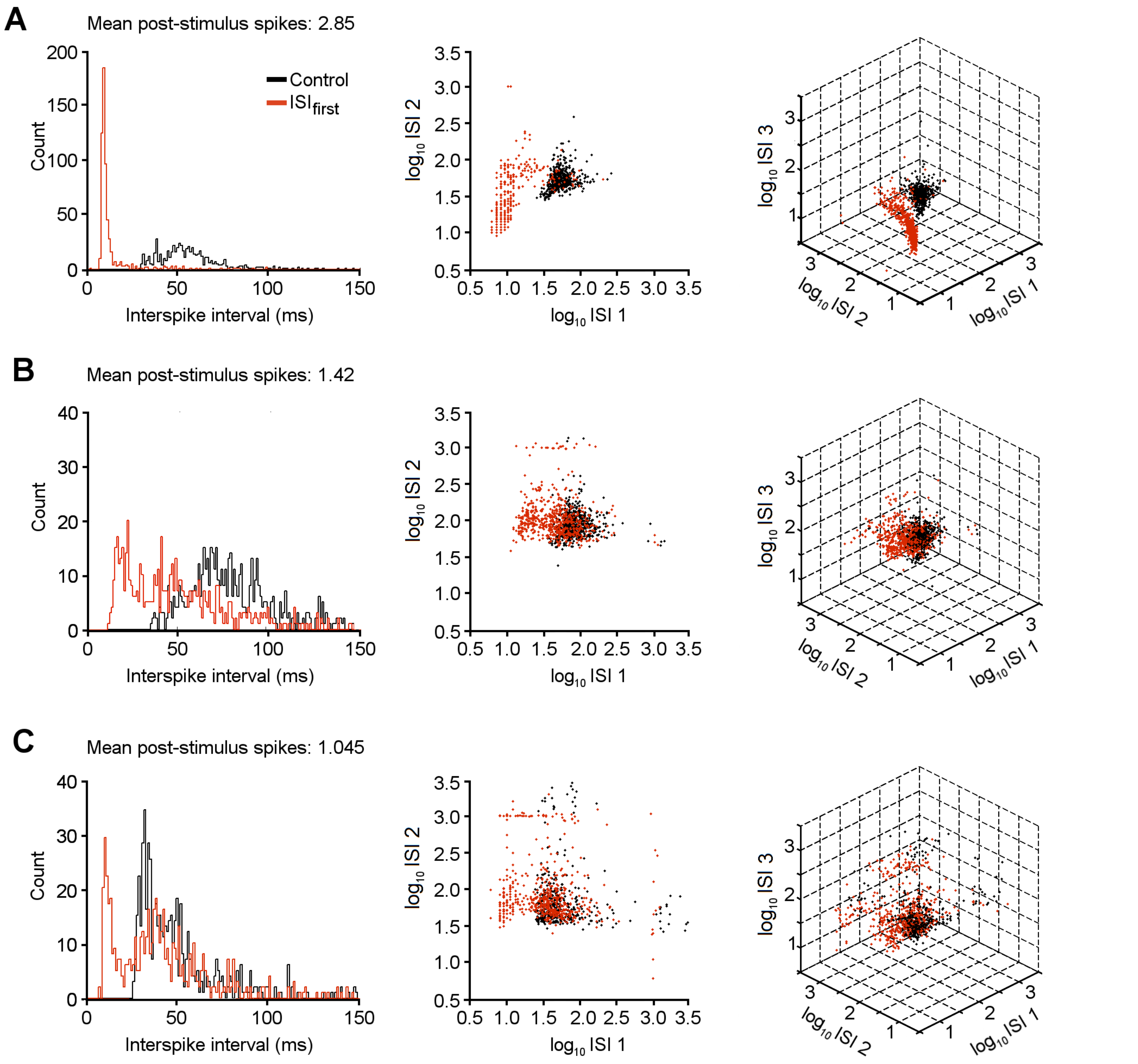

Figure 8. Post-stimulus spike train structure. A) Interspike intervals for a cell stimulated at 1 Hz and 25 μA in the molecular layer, with mean post-stimulus spike number of 2.85. Left panel shows histogram of first ISI (red line) and spontaneous spikes (black line). Middle panel shows a plot of second ISI against first ISI, for post-stimulus spikes (red dots) and spontaneous spikes (black dots). Note that separation between populations is evident on x axis but less so on y axis. Right panel shows plot of third ISI, against second and first ISIs. Again, no separation is evident on third axis. B) Similar plots to panel A, for cell with mean post-stimulus spike number of 1.42. C) Similar plots to panels A and B for cell with mean 1.045. Note there is no clear clustering of evoked spikes in B or C that would allow stimulus-specific spike patterns to be distinguished from spontaneous activity.
doi:10.1371/journal.pone.0087828.g008

robust (Fig. 3F), as the decrease in ISI with increasing stimulation intensity did not cover a very wide dynamic range, but under conditions where several spikes are added (e.g. in the absence of feedforward inhibition) the relationship is clearer [8].

Purkinje Neuron Firing in vivo

Our results suggest that couplets should appear on the Purkinje neuron output train even when granule neurons inputs fire at low frequencies. It has long been known that sensory stimulation results in obvious bursts of high-frequency discharge from Purkinje neurons. This follows from the fact that most granule neurons tend to fire in high frequency bursts – hundreds of hertz – which would result in the straightforward relay of the burst onto the output train of the Purkinje cell, even if each input pulse only generates a single simple spike in the Purkinje cell (see Fig. 2A and 2B for the *in vitro* equivalent). However, considerable heterogeneity in granule neuron firing rates is known at rest and during stimulation, apparently varying with the strength of cutaneous input and position within the granular layer [12]. Those granule neurons firing regularly at frequencies less than ~30 Hz would be adding spikes at rates less than or equal to the mean rate of intrinsic firing in the Purkinje cell. This presents the greatest challenge for signal

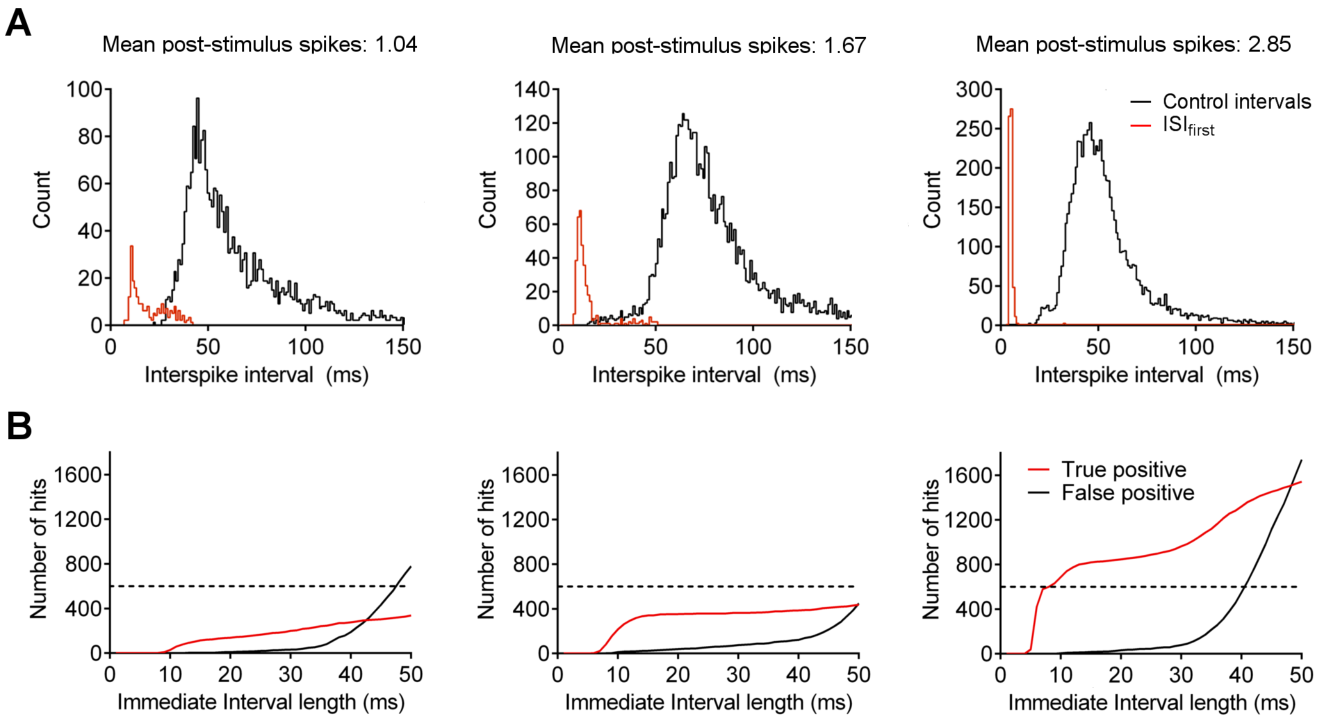

Figure 9. Predictive power of couplet motif. A) Distribution of first post-stimulus intervals (ISI_{first}, red line) and interspike intervals uncorrelated with stimuli (black line), for three representative cells with mean post-stimulus spike numbers of 1.04, 1.67 and 2.85 (as indicated). Note intervals > 150 ms have been omitted for clarity. B) Plot of the number of stimuli that were correctly detected by the couplet motif (true positives, red line) and the number of couplet motifs assigned incorrectly to spontaneous events (false positives, black line), against the interval width used to define the couplet (immediate interval length). Total number of stimuli in all cases was 600 (dashed line).
doi:10.1371/journal.pone.0087828.g009

discrimination: input signals arriving at a rate similar to background activity. To confound analysis even further, such stimuli would represent a small fraction of the total spike population (see Fig. 7), and would also be swamped by high-frequency inputs. As such, confident identification of these signals in a population histogram would be hard, and so despite evidence for apparent subpopulations of short ISIs being recognisable in some reports (e.g. see Fig. 4 in [20]; Fig. 1 in [4]), the origin of these spikes cannot be directly attributed to couplets rather than high-frequency input trains. However, if a couplet motif was imposed by these low-frequency inputs, the cell would be able, in principle, to recognise the stimulus as distinct from intrinsic spiking, on a moment to moment basis.

Potential Physiological Roles of Couplets

The ability to discriminate stimulus events from background firing would only have computational value if neurons are equipped with a physiological mechanism for decoding the couplet pattern. The Purkinje neuron axons form an inhibitory connection with deep cerebellar nuclear (DCN) neurons [21], and this connection exhibits depression at high frequencies [22,23]. The relationship between input rate and firing rate in DCN neurons is complex, however, in that phasic depression of IPSP amplitude during 100 Hz stimulation is counterbalanced by a build up of a tonic IPSP [22]. A couplet of pulses would give a mixed outcome: tonic IPSP amplitude would be increased for the second pulse, but phasic IPSP amplitude would be decreased. The likely outcome would be a transient increase in DCN inhibition due to tonic summation, which would recover with the time course of tonic IPSP decay.

In addition to this downstream effect, an alternative decoding mechanism may reside in the Purkinje neuron itself. Action potentials originate in the proximal section of the axon, and propagate back into the cell soma, and less effectively into the dendritic tree [24,25]. Speculatively, the generation of rapid sequential depolarisations at the cell soma associated with a couplet may lead to summation of voltage-dependent signalling processes that modulate Purkinje cell function. High amplitude calcium concentration changes in the soma due to rapid summation of calcium influx through voltage-operated calcium channels would be an example of such a process, and may engage effectors within the cytoplasm and nucleus. Given that couplets do not appear to arise from intrinsic spiking mechanisms, these changes in somatic calcium signalling may provide a mechanism for downstream targets to be engaged selectively by synaptic inputs.

In summary, we have demonstrated the utility for signal discrimination of parallel fibre inputs evoking couplets of action potentials in Purkinje cells. Whether this theoretical capacity has physiological meaning awaits further research.

Acknowledgments

We thank Julie March for technical assistance with slice preparation.

Author Contributions

Conceived and designed the experiments: KR NR TB. Performed the experiments: KR NR TB. Analyzed the data: KR NR TB. Wrote the paper: KR NR TB.

References

1. Hausser M, Clark BA (1997) Tonic synaptic inhibition modulates neuronal output pattern and spatiotemporal synaptic integration. Neuron 19: 665–678.
2. Raman IM, Bean BP (1999) Ionic currents underlying spontaneous action potentials in isolated cerebellar Purkinje neurons. J Neurosci 19: 1663–1674.
3. Williams SR, Christensen SR, Stuart GJ, Hausser M (2002) Membrane potential bistability is controlled by the hyperpolarization-activated current I(H) in rat cerebellar Purkinje neurons in vitro. J Physiol 539: 469–483.
4. Yartsev MM, Givon-Mayo R, Maller M, Donchin O (2009) Pausing purkinje cells in the cerebellum of the awake cat. Front Syst Neurosci 3: 2.
5. Engbers JD, Anderson D, Asmara H, Rehak R, Mehaffey WH, et al. (2012) Intermediate conductance calcium-activated potassium channels modulate summation of parallel fiber input in cerebellar Purkinje cells. Proc Natl Acad Sci U S A 109: 2601–2606.
6. Loewenstein Y, Mahon S, Chadderton P, Kitamura K, Sompolinsky H, et al. (2005) Bistability of cerebellar Purkinje cells modulated by sensory stimulation. Nat Neurosci 8: 202–211.
7. Mittmann W, Hausser M (2007) Linking synaptic plasticity and spike output at excitatory and inhibitory synapses onto cerebellar Purkinje cells. J Neurosci 27: 5559–5570.
8. Walter JT, Khodakhah K (2006) The linear computational algorithm of cerebellar Purkinje cells. J Neurosci 26: 12861–12872.
9. Walter JT, Khodakhah K (2009) The advantages of linear information processing for cerebellar computation. Proc Natl Acad Sci U S A 106: 4471–4476.
10. De Schutter E, Steuber V (2009) Patterns and pauses in Purkinje cell simple spike trains: experiments, modeling and theory. Neuroscience 162: 816–826.
11. Chadderton P, Margrie TW, Hausser M (2004) Integration of quanta in cerebellar granule cells during sensory processing. Nature 428: 856–860.
12. Jorntell H, Ekerot CF (2006) Properties of somatosensory synaptic integration in cerebellar granule cells in vivo. J Neurosci 26: 11786–11797.
13. van Kan PL, Gibson AR, Houk JC (1993) Movement-related inputs to intermediate cerebellum of the monkey. J Neurophysiol 69: 74–94.
14. James LR, Andrews S, Walker S, de Sousa PR, Ray A, et al. (2011) High-throughput analysis of calcium signalling kinetics in astrocytes stimulated with different neurotransmitters. PLoS One 6: e26889.
15. Mittmann W, Koch U, Hausser M (2005) Feed-forward inhibition shapes the spike output of cerebellar Purkinje cells. J Physiol 563: 369–378.
16. Marcaggi P, Attwell D (2007) Short- and long-term depression of rat cerebellar parallel fibre synaptic transmission mediated by synaptic crosstalk. J Physiol 578: 545–550.
17. Engbers JD, Fernandez FR, Turner RW (2013) Bistability in Purkinje neurons: Ups and downs in cerebellar research. Neural Netw 47: 18–31.
18. Steuber V, Mittmann W, Hoebeek FE, Silver RA, De Zeeuw CI, et al. (2007) Cerebellar LTD and pattern recognition by Purkinje cells. Neuron 54: 121–136.
19. Jorntell H, Bengtsson F, Schonewille M, De Zeeuw CI (2010) Cerebellar molecular layer interneurons - computational properties and roles in learning. Trends Neurosci 33: 524–532.
20. Jaeger D, De Schutter E, Bower JM (1997) The role of synaptic and voltage-gated currents in the control of Purkinje cell spiking: a modeling study. J Neurosci 17: 91–106.
21. Uusisaari M, De Schutter E (2011) The mysterious microcircuitry of the cerebellar nuclei. J Physiol 589: 3441–3457.
22. Telgkamp P, Raman IM (2002) Depression of inhibitory synaptic transmission between Purkinje cells and neurons of the cerebellar nuclei. J Neurosci 22: 8447–8457.
23. Zheng N, Raman IM (2010) Synaptic inhibition, excitation, and plasticity in neurons of the cerebellar nuclei. Cerebellum 9: 56–66.
24. Stuart G, Hausser M (1994) Initiation and spread of sodium action potentials in cerebellar Purkinje cells. Neuron 13: 703–712.
25. Clark BA, Monsivais P, Branco T, London M, Hausser M (2005) The site of action potential initiation in cerebellar Purkinje neurons. Nat Neurosci 8: 137–139.
